# Gut Microbiota and Iron: The Crucial Actors in Health and Disease

**DOI:** 10.3390/ph11040098

**Published:** 2018-10-05

**Authors:** Bahtiyar Yilmaz, Hai Li

**Affiliations:** 1 Maurice Müller Laboratories, Department of Biomedical Research, University of Bern, 3008 Bern, Switzerland; hai.li@dbmr.unibe.ch; 2 University Clinic of Visceral Surgery and Medicine, Inselspital, 3010 Bern, Switzerland

**Keywords:** iron, gut microbiota, iron supplementation, iron transporters, mucosal immunity, SCFA, intestinal inflammation, inflammatory bowel disease (IBD), colorectal cancer

## Abstract

Iron (Fe) is a highly ample metal on planet earth (~35% of the Earth’s mass) and is particularly essential for most life forms, including from bacteria to mammals. Nonetheless, iron deficiency is highly prevalent in developing countries, and oral administration of this metal is so far the most effective treatment for human beings. Notably, the excessive amount of unabsorbed iron leave unappreciated side effects at the highly interactive host–microbe interface of the human gastrointestinal tract. Recent advances in elucidating the molecular basis of interactions between iron and gut microbiota shed new light(s) on the health and pathogenesis of intestinal inflammatory diseases. We here aim to present the dynamic modulation of intestinal microbiota by iron availability, and conversely, the influence on dietary iron absorption in the gut. The central part of this review is intended to summarize our current understanding about the effects of luminal iron on host–microbe interactions in the context of human health and disease.

## 1. Introduction

The availability of iron is enormously vital for many living organisms, particularly humans and microbes. Iron has a direct impact on host–microbiota interactions via altering microbial/viral growth, acting on the host immune system, and drafting in a range of biochemical processes critical to sustain life [1,2,3]. Most living beings have evolved to acquire iron from their proximate niche as an evolutionary conserved strategy. Iron mainly works as an universal co-factor for proteins such as hemoglobin, and for numerous enzymes involved in oxygen transport mechanisms, mitochondrial respiration, intermediary and xenobiotic metabolism, and fundamental biological processes such as cell growth and differentiation [4]. Nonetheless, iron deficiency, the most prevalent nutritional disorder, or iron overload in gut due to its malabsorption, can alter host mucosal immune responses. Notably, this is supported by several observations in the course of infectious disease or intestinal inflammatory disease [3,5,6]. Conversely, an accumulated body of evidence also suggests that immune activation can regulate iron metabolism that then leads to the development of iron-restricted anemia [1,5,7,8]. In this review, we meticulously cover the multifaceted aspects involved in iron-mediated host–microbe interactions in the gut, for a better understanding of bi-directional cross-talk between iron homeostasis and the mucosal immune system primed by gut microbiota. We begin with introducing general concepts of gut microbiota and metabolic stress in gut lumen. We then concisely present systemic iron metabolism and homeostasis concepts. The central part of this review focuses on our current knowledge about mechanisms mediating the effects of luminal iron on host intestinal immune responses, as well as the effects of abnormal gut immunity on iron homeostasis due to changes in abundance of commensal and pathogenic bacteria in gut. We last discuss the effects of iron metabolism on intestinal inflammation and colorectal cancers via modulation of the gut microbial profile.

## 2. Mammalian Gut Microbiome in Health

Humans and other animals co-exist with vast numbers of microorganisms in their lower intestine, and they are in continuous interaction with these entities on a daily basis. If one thinks of a human as a host–microbial super-organism, these prokaryotic constituents comprise 90% of our total cells and contain 99% of the aggregate gene pool [9]. The existence of highly co-evolved mutualism between microbes that inhabit body surfaces and the host immune system have promoted beneficial co-existence and interdependency over millions of years. Such mutualism starts at birth and continues throughout life, driven by the colonization of microbial consortia within specific niches. Mucosal surfaces are densely colonized by bacteria, fungi, archaea, viruses, and parasites that are mainly non-pathogenic in healthy hosts: the extended metabolic potential of biochemical pathways in microbes crucially contribute to host physiology, including digestive [10,11] and protective [12,13,14,15] functions, microbial catabolism of otherwise indigestible foodstuffs [16], provision of essential amino acids, maturation of host mucosal immune system [17,18,19,20], and completing the bile-salt cycle and pre-systemic metabolism of drugs and toxins [21,22,23,24,25,26]. By far, the gastrointestinal tract (GI) is the most heavily colonized organ in humans, and it contains over 70% of all the microbes in the body. The human gut has an estimated surface area of a tennis court, and it is a preferred site for colonization due to its constant physiological temperature and richness in molecules that can be used as nutrients by microbes. Though bacteria belonging to Bacteroidetes (~16–23%) and Firmicutes (~49–76%) phyla, and to a lesser extent, Actinobacteria (<5%) and Proteobacteria (<10%) constitute the main players in human intestines, besides, there is a greater diversity at lower taxonomic levels. Prominently, the viable intestinal microbiota are critical for retaining a healthy host [27]. However, host–microbial interactions are not always mutualistic; unfortunately, like any beautiful relationship, this mutualism can also turn sour [26,28]. Several features of the modern lifestyle directly contribute to this situation via antibiotics and other medications, including birth control and non-steroidal anti-inflammatory drugs, diets high in refined carbohydrates, sugar, and processed foods or low in fermentable fibers, dietary toxins such as gluten in wheat and industrial seed oils, and the modern plague chronic stress. Under these extreme pathophysiological conditions, the interactions can be subsumed in a pathogenic relationship, leading to alterations in the composition of microbial consortia and their metabolic functions, accompanied by a loss of fitness of the host—producing the occurrence or manifestation of disease [11], including many gastrointestinal disorders such as diarrhea, gastroenteritis, irritable bowel syndrome (IBS), and inflammatory bowel disease (IBD) [29,30,31,32,33]. However, the uncharacterized features of different prokaryotic constituents within the diverse microbiological environment that can provoke different types of host immune responses that still make it difficult to identify the source(s) of a soured mutualistic relationship. 

Many characteristics concerning mammalian gut microbiota, including the dynamics impact of its assembly, which define the spatial distribution and functional features of its prokaryotic members, remain vague. Concurrently, the factors involved in shaping the gut microbiota were extensively studied in the last decade. Well-characterized factors that influence gut colonization during life are among diet (including breast feeding and formula-based feed in early life), hygiene, illness, medication, surgery, hospitalization, stress, sport activity, aging, and smoking and alcohol abuse, which all can be classified as environmental factors [34,35]. Even though gut microbial changes can partially be explained by host genetics [36,37,38], a recent study shows inter-individual gut similarities in the gut microbial profiles of genetically unrelated individuals sharing a household pattern, and that over 20% of the inter-individual microbiome variability is associated with environmental factors such as diet and medication [35]. Interestingly, this study additionally demonstrates that there is limited evidence for micro biome–genetic associations, based on an analysis performed on a cohort of 1046 healthy adults [35]. Even though there are minor heritable taxa and SNP associations, gut microbial composition is predominantly shaped by non-genetic factors [39,40,41]. Gaining mechanistic insight into the regulation of host–microbe interactions and the development of microbial consortia within a specific niche is of fundamental importance for discriminating the associations and causalities between the intestinal ecosystem and host immunity. This will undoubtedly lay the foundation for the future therapies of intestinal inflammation-linked diseases [26].

## 3. Systemic Iron Metabolism and Homeostasis

A healthy human can absorb 25–50 g of dietary iron over lifetime. The majority of body-constituent iron (~3–5 g) is presented as heme, an iron-containing compound of the porphyrin class in the hemoglobin of red blood cells (RBCs), or in the myoglobin of muscles [42]. In order to replace iron losses through urine, sweat, and desquamated enterocytes, humans are able to absorb iron in a daily basis. On average, 2 mg of iron is delivered by dietary absorption into the duodenum, which is balanced by an unregulated loss of 2 mg of iron. Dietary iron has three forms: inorganic, heme, and ferritin. Inorganic dietary iron, existing in almost all diet sources, is mainly present in the oxidized form, Fe(III), and this needs to be reduced to the Fe(II) form via ferrireductases prior to intestinal uptake [43,44]. Although heme mainly derived from lean meat accounts for only 5–10% dietary iron, it is more readily available compared to non-heme iron. Even though the uptake of dietary heme and ferritin mechanistically is not well identified, evidence suggests that iron is consequently released from these forms, and it enters a common pathway in the enterocyte as inorganic iron. The circulation of iron is relatively small, and it must have a turnover of few hours to meet the daily requirement of iron to support normal body functioning. The balance of iron level in human body is extremely important, and since humans do not have a physiological mechanism for iron excretion, intestinal iron absorption is a highly regulated dynamic process. Players such as macrophages in the spleen, liver, and bone marrow maintain a transient fraction of iron, while an excess of the metal is stored in the liver parenchyma within ferritin [45,46]. Despite rapid turnovers and changes in host iron utilization, plasma iron concentration is generally stable, indicating that the delivery of iron from recycling macrophages into plasma is homeostatically controlled. Iron is an essential bio-element for most life forms, and its importance lies in its ability to mediate electron transfer (The ferrous state of iron acts as an electron donor, and its ferric state acts as an acceptor). Therefore, iron plays a vital role in the catalysis of enzymatic reactions that involve electron transfer (reduction and oxidation, redox reaction). Even though it is a critically essential micronutrient, in reverse, it is a deleteriously toxic oxidative radical when allowed to exchange electrons in an unrestrained manner with hydrogen peroxide (H_2_O_2_), which it leads into the production of hydroxyl radicals and hydroxide ions via Fenton chemistry. Hence, the balance between deficient or excessive levels of iron can be harmful for the host via damage to DNA, protein, and lipids [47]. Therefore, this balance is tightly regulated at the systemic and cellular levels by two distinct but interacting sets of regulatory mechanisms that humans and other organisms, therefore, evolved to have [4,42,48]. 

**Figure 1:**
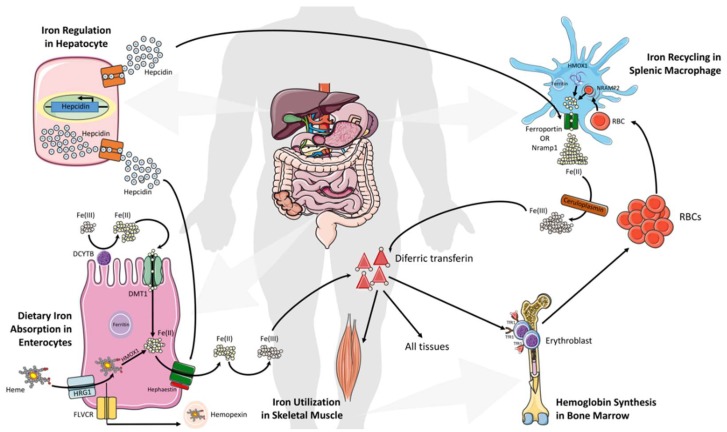
Systemic iron metabolism. Cells and organs involved in iron regulation are shown. Hepcidin produced in hepatocytes regulates iron efflux from other cells by regulating the stability of ferroportin. Hepatocytes sense iron levels and release hepcidin accordingly. Divalent metal transporter 1 (DMT1) on enterocytes internalize iron from the lumen of the duodenum after ferric Fe(III) is reduced to ferrous Fe(II) by ferrireductase. In parallel, free heme is internalized via HRG1 and hemoxygenase-1 (HMOX1) helps to release Fe(II). Ferroportin on the enterocyte’s membrane that cooperates with hephaestin (HEPH) oxidizes Fe(II) to Fe(III). Besides, hepcidin binds to ferroportin on macrophages and duodenal enterocytes and splenic reticuloendothelial macrophages recycle iron from senescent red blood cells and release via ferroportin with the aid of natural resistance-associated macrophage protein 1 (Nramp1). Fe(II) is then oxidized into Fe(III) via ceruplasmin (Cp) in the circulation. Plasma transferrin (Tf) captures and circulates iron in the body, and Tf–Fe_2_ supplies iron to all tissues in host body. Hepatocytes sense iron levels in host and release hepcidin, a hepatic hormone that regulates iron efflux from these cells by regulating the stability of ferroportin. The synthesis and secretion of hepcidin by hepatocytes is also influenced by several conditions in the host, including inflammation, endoplasmic reticulum (ER) stress, and hypoxia.

The uptake of all forms of iron occurs mainly in the duodenum and upper jejunum. Systemically, duodenal enterocytes absorb inorganic dietary non-heme ferric iron via divalent metal transporter 1 (SLC11A2 or DMT1) after reduction by membrane bound ferrireductases (DCYTB), the enzymes that reduce ferric iron to ferrous iron, often as a by-product of another operation (Figure 1). Iron can also adopt different spin states (high or low) in both the ferric and ferrous form, depending on its ligand environment. Enterocytes are also able to uptake heme iron via an undefined mechanism (however, the proposed transporter SLC46A1 in this study then appears to carry mostly folate) [49,50]. Iron translocation at the cellular level occurs through the enterocytes and is exported into circulation by the basolateral exporter ferroportin (SLC40A1) via a mechanism dependent on the oxidation of iron by a membrane-bound multi-copper oxidase hephaestin enabling binding between plasma transferrin (Tf) and iron. Most cells in the human body obtain iron from circulating diferric Tf (Tf-Fe(III)). This key form binds to transferrin receptor 1 (TfR1), which is highly expressed on hemoglobin-synthesizing erythroblast cell surfaces and is internalized as a Tf-Fe(III)–TfR1 complex by endocytosis. Later, ferric iron is released from Tf upon acidification of the endosomes, and this is followed by reduction via STEAP3. Upon the reduction, it is exported into the cytosol by DMT1. This cytosolic form of iron is used then for the formation of iron-containing proteins and by the mitochondria for the biosynthesis of heme and Fe–S clusters [51]. When enough iron is stored in the human system, iron export is reduced via hepcidin (a 25-amino acid peptide hormone)-mediated internalization and the degradation of ferroportin. Additionally, ferritin stores iron, which can be lost within three days by intestinal cells shedding (Figure 1) [51,52]. 

Daily absorbed iron (1–3 mg) represents only a fraction of the total body iron, while the recycling of heme from senescent erythrocytes by reticuloendothelial (RE) macrophages provides the main fraction of circulating iron [53]. Ferroportin exports the iron from heme into the circulation, and binds to apotransferrin for hemoglobin synthesis in the bone marrow. However, liver hepatocytes play a critical role in regulating serum iron levels via the integration of information on the systemic iron status, and secreting an appropriate amount of hepcidin that orchestrates systemic iron fluxes and controls plasma iron levels (Figure 1) [4,54]. Hepcidin also influences the internalization of ferroportin, decreasing iron export. An Increased level of hepatic iron (>30 μmol/g of dry weight) and inflammation are positively correlated with hepcidin production, and they are negatively correlated with ferroportin degradation in intestinal cell RE macrophages, which leads to an iron reduction in plasma [55,56]. Mechanistically, iron–transferrin complexes bind to TfR1 on hepatocytes, thereby displacing the TfR1-associated protein, HFE. Then, the binding interaction between HFE and hepatocyte-specific type 2 transferrin receptor (TfR2) transduces signals acting together with other signals from bone morphogenetic proteins (BMPs) to increase hepcidin secretion. This leads the binding of hepcidin to the transporter ferroportin on enterocytes and macrophages to induce its internalization and lysosomal degradation, thus reducing the entry of iron into the circulation and restoring homeostasis (Figure 1) [1,8]. In contrast, low levels of plasma iron control the inhibition of hepcidin expression and an increase in transporter ferroportin, which allow more iron into the blood circulation [1,8]. Of note, perturbations in hepcidin production, either inherited or acquired, consequently trigger iron deficiency (high hepcidin levels) or iron overload (hepcidin deficiency).

## 4. Iron Regulation Along the Gastrointestinal Tract (GIT) Under the Shade of the Gut Microbiota

**Figure 2:**
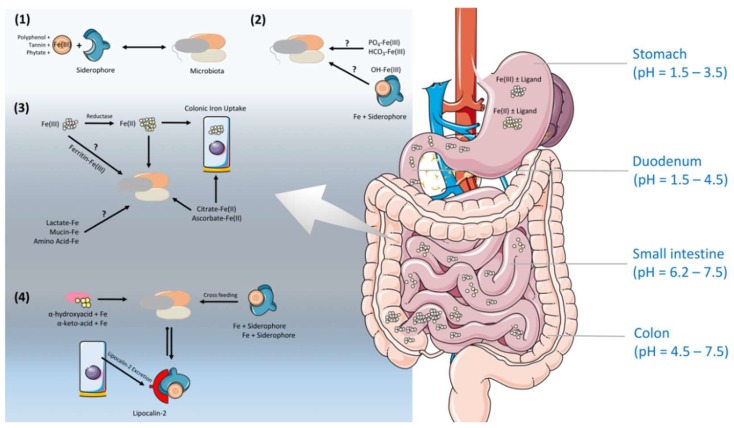
Several iron regulation mechanisms in the colonic lumen. The pH varies along the gastrointestinal tract (GIT), and food intake can also drive further pH fluctuations in the GIT. The stomach has a low pH (pH = 1.5–3.5) that favors the solubility of both ferric and ferrous iron with or without a ligand. Even though the pH is low in the duodenum (pH = 1.5–4.5), the acidic nature of the environment, mixed with food components, can increase the pH. A higher pH in the small intestine (pH = 6.2–7.5) decreases the solubility of ferric iron, and within the colon, the pH can slightly drop due to lactate and short chain fatty acids (SCFAs; acetate, butyrate, and propionate) produced by the microbiota (pH = 4.5–7.5). In colonic lumen, (**1**) iron can bind to polyphenols, including tannins and phytate, that can make iron accessible via the enzymatic degradation or removal of the iron by siderophores; (**2**) An insoluble form of iron with phosphate, carbonate, or oxides can be made soluble again via as-yet unidentified mechanisms that drive bacterial reduction or siderophore chelation; (**3**) Host cells and/or gut microbes can utilize the reduced form of iron conjugated with citrate or ascorbate, and additionally, iron-bound lactate, mucin, or amino acids might be easier to access compared to an iron−ferritin complex by colonic microbiota via unknown mechanism(s); (**4**) The low-affinity siderophores, alpha-hydroxyacids and alpha-keto-acids may theoretically assist with the relatively easier access of iron, and they may also help for the iron cross-feeding by heterologous siderophores (a phenomenon where certain bacterial strains can compete for each other’s siderophores) within the colonic microbiota. At last, lipocalin-2 in the colonic lumen may scavenge iron conjugated to siderophores to prevent uptake by pathobionts.

The stomach is an oxygenic and acidic environment [57]. The nature of diets and the stomach leads the most of the dietary iron to reach the intestine in ferrous form, Fe(II), assisted by reducing agents, such as ascorbic acid [57,58]. Contrary to that, in the small intestine, the pH rises, and hence, the solubility of ferric iron decreases and the oxidation of iron increases [59]. Several studies demonstrate the role of colonic microbiota on this iron, with a shift in the valence state and the importance of siderophore production (Figure 2) [60,61,62,63]. Nevertheless, the iron solubility and availability in the colonic lumen for gut microbiota is extremely difficult to predict, due to the direct/indirect influence of many environmental and conditional factors. Depending on the dietary availability, only ~15% of iron is absorbed in the duodenum, the primary site of iron absorption, and the remainder passes into the colon, where it is available for utilization by the gut microbiota. Despite a relative high theoretical concentration (~25 mmol/L) of iron presenting in the large intestine, only a small proportion (~0.4 mmol) is bioavailable, likely due to the limited water solubility of inorganic iron in a non-acidic microenvironment [64]. Additionally, iron transporters such as DMT1 have been shown to express in the apical surfaces of the mammalian proximal colon, indicating an involvement of the host in exacerbating the iron availability in the bacteria-dense large intestine [65,66]. Iron speciation and the potential presence of lactoferrin, also known as lactotransferrin, lipocalin-2 (only expressed at low level in healthy host) and as-yet unidentified defence proteins in colonic mucosa might contribute to the limitation of iron at this site, which enables gut microbes to synthesize siderophores, the small, high-affinity iron-chelating compounds, for their needs under the circumstances of limited amount of iron in their surrounding environment (Figure 2) [67].

Not only oxygen and pH, but also different dietary products can also affect the valency and the solubility of iron. Certain dietary products, mainly derived from plant sources including phytate [68,69], polyphenols [70], and tannins [69] negatively affect iron absorption by tightly binding to iron and decreasing iron bioavailability. Vitamin C is a water-soluble vitamin that is thought to increase the absorption of non-heme iron, and it acts as a reducing agent to facilitate iron absorption from the GIT [71,72]. Other organic acids such as tartaric, malic, succinic, fumaric, and citric acids can prevent the precipitation of ferric iron when the pH increases, and this enhances Fe(II) and Fe(III) uptake [71,73]. Moreover, the fluctuations in gut metabolites cause an increase in short-chain fatty acids (SCFAs), which can lower the pH, promote solubility, and reduce iron into the ferrous state, and importantly, via stimulating the proliferation of epithelial cells, enhance the absorptive surface [63]. However, the efficiency of colonic iron absorption is only about 14% that of the duodenum. The expression of several critical genes in iron absorption pathway, including Dcytb, DMT1, TfR, and ferritin, are lower (not ferroportin) in the colon than in the duodenum [74,75]. In contrast, colonic epithelial cells express basolateral IREG1 in the same fashion as in the duodenum, and this protein could regulate colonic epithelial cell iron levels [60]. Mice studies clearly showed that iron absorption genes in the colon are up-regulated compared to iron-deficient mice, whereas Dcytb (a highly expressed duodenal reductase) is down-regulated [60]. This hints at the influential role of the colonic microbiota on the valence state of iron, by acting on extracellular reductases (Figure 2). A recent study indicates a direct role of host microbiota in iron regulation. The study reported a 10-fold increase in intestinal Dcytb and Dmt1 expression, and a two-fold reduction in ferroportin expression in germ-free (GF) mice, as compared to specific pathogen free (SPF) mice [76]. Therefore, in the absence of gut microbiota, the intestinal cells displayed very low iron stocks, and transport systems towards the body were very scarce. However, in the presence of gut microbiota, these cells acquired a considerable capacity for iron storage (in the form of ferritin), and favored its transport towards the body by increasing the expression of ferroportin. This shows that intestinal cells have a capacity to adapt their ability to distribute and store iron in the presence of gut microbiota. This notion is further supported with GF studies in rats, showing that the reduced level of iron uptake increased the loss of iron in their feces compared to specific-pathogen-free (SPF) rats [77], and they become anemic when fed on a low-iron diet [77]. The authors estimated that the absorption and net retention of iron decreased by around 25% in the absence of viable intestinal microbiota [77], in agreement with other studies that found a decreased absorption of iron after antibiotic treatment in rats [78] and rabbits [79]. Additionally, elevated ferritin expression and epithelial cells favoring iron storage upon gut colonization in GF mice provide an insight that gut microbes can establish a specific iron regulation signature for crosstalk with the host intestinal epithelium. Notably, due to the reduced environment in the colonic lumen, iron can form complex formations with mucins, certain amino acids, proteins, and other food components. However, we do not entirely know yet how accessible these insoluble forms of iron are for bacteria [80]. Somehow, ferrous and ferric forms of iron are be present in the colonic lumen to favor the viability of gut microbiota.

We have more information on how the mammalian host cells in the gut are able to deal with iron; however, we are quite restricted on the roles of the gut microbiota on iron regulation, which remains speculative [65]. Iron availability for small intestinal microbiota, explicitly in the duodenum, are likely to be different to that for colonic microbiota, since small intestinal microbiota are home to a lower density of residing microorganisms compared to the colon. Nevertheless, colonic iron absorption can contribute more to defence mechanisms, as iron exclusion from the colonic lumen can contribute to nutritional immunity and restrain the gut pathobiont community [81]. Of note, oral iron administration can modify gut microbiota due to metabolic changes in the colonic lumen. 

## 5. The Effect of Iron on Gut Microbiota and Pathogens

The human gut microbiota encounters a broad range of unabsorbed luminal iron concentrations acquired via a diet containing red meat and fortified cereals. Iron as an essential element, is also extensively required across the domain of bacteria by functioning as a co-factor in iron-containing proteins for redox reaction, metabolic pathways, and electron transport chain mechanisms [82,83]. These gut residents, just like humans, have evolved a number of mechanisms for obtaining iron from their human hosts for survival and proliferation. 

Iron is critical for the replication and survival of almost all bacteria, with a few exceptions, which acquired alternative metabolic solutions from evolution. *Lactobacillus plantarum* was the first identified iron-independent microbial strain, which contains just one or two iron atoms—a level that is considered to be too low to provide iron with any conceivable biological function [84]. This feature also explains their presence in natural gut microbiota and milk, a highly iron-restricted environment due to the lactoferrin [85]. Another novel microorganism is *Borrelia burgdorferi*, a well-known pathogen causing Lyme disease transmitted to humans by the bite of infected ticks of the genus *Ixodes*. This pathogen have evolved in an iron-poor but a manganese-rich environment, by substituting Fe with Mn in their metalloproteins, which is an essential trigger for the activation of SodA superoxide dismutase (SOD), and which is essential for virulence [86]. This may facilitate infection in iron-free conditions that is tightly restricted within the host systemic compartment [87].

Alternatively, siderophores are small, high-affinity iron-chelating compounds that are secreted by bacteria, and they are the most prevalent strategies of aerobic and facultative anaerobic bacteria families such as Enterobacteriaceae, Streptomycetaceae, and Bacillaceae, in order to scavenge inorganic iron from the environment [88]. They are vastly produced by bacteria under low iron stress, due to their high ferric ion-specific chelating capacities [83,89]. There is no shared protein structure of siderophores due to the ability of the gut bacterial species to produce iron-siderophore complexes with specific transporters [88]. 

On the other hand, some gut strains like *Bacteroides fragilis* are strongly dependent on heme (or its precursor, protoporphyrin IX), since they have dispensed with the biosynthetic machinery that is required for heme elaboration. Microbes can take up heme by releasing either hemophores or expressing high-affinity heme outer membrane transporters [90]. In iron depletion, heme availability in the GIT is likely to be limited [91]. Thus, iron availability severely influences the gut bacterial ecosystem. Not surprisingly, different studies have investigated the effect of iron deficiency and/or supplementation on shaping the composition of the intestinal microbiota, both in animals and humans. These studies revealed well-defined patterns of microbial alterations in the gut which correlate with iron-deficient and iron-supplemented diets. 

**Figure 3:**
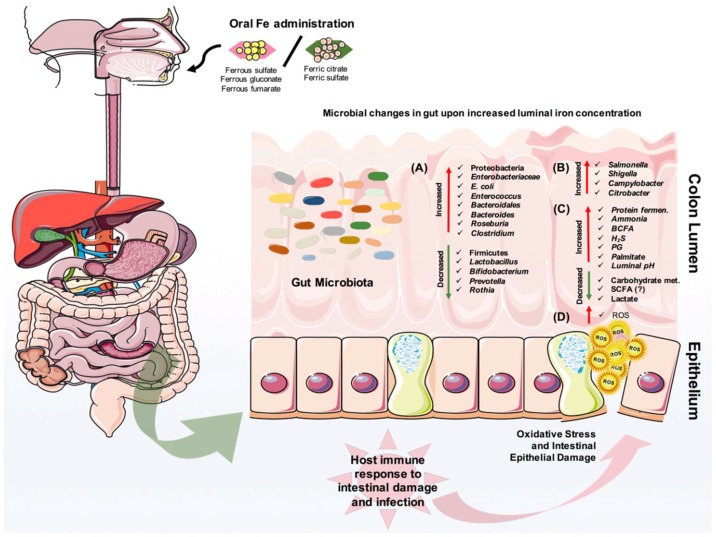
Microbial and metabolic changes in the colonic lumen after oral iron administration. Orally administered iron has a direct impact on alteration of microbial composition in the gut. It can result in reduction in the beneficial microbiota and the expansion of pathobionts (**A**), and this can also provide an opportunity for the expansion of enteric pathogens (**B**). The host metabolism is additionally influenced with an increase in protein fermentation and reduction in carbohydrate metabolism (**C**). Importantly, iron can induce the generation of reactive oxygen species (ROS) in the gut (**D**), which causes oxidative stress and consequently, intestinal epithelial damage. In turn, the host intestinal immune system responds with inflammation, intestinal damage, and possible infection.

Numerous studies have investigated the effect of iron deficiency and supplementation on the gut microbiota (summarized in Figure 3). One of the oldest studies back in 1985, showed that infants given an iron-fortified cow’s milk preparation had lower *Bifidobacterium* but higher counts of *Bacteroides* and *E. coli* than infants receiving an unfortified cow’s milk preparation [92]. Another study on prolonged consumption of iron-supplemented biscuits by children from Côte d’Ivoire demonstrated a high proportion of fecal *Enterobacteriaceae* family and a low proportion of *Lactobacillus*, compared to a control group receiving non-supplemented biscuits [93]. Moreover, iron deficiency in young Indian women was associated with low levels of *Lactobacillus acidophilus* in the gut [94]. In a recent study, an iron-fortified micronutrient powder provided to Kenyan infants ranging from 6 to 10 months of age caused an increase of several taxa from *Enterobacteriaceae* family, especially the pathobiont *E*. *coli*, and a decrease of *Bifidobacterium* in their intestine [95]. Of note, the researchers also stated on higher levels of calprotectin in infants supplemented with iron, an indication of increased gut inflammation [95]. A lack of host factors such as iron status, immune system, and diet fluctuation in the gut might be drawbacks to studying iron and microbiota. Nevertheless, in vitro studies hint on microbial metabolism in the presence of iron and nutrients. An in vitro colonic fermentation study using immobilized human fecal microbiota to show the impact of Fe deficiency and sufficiency showed that during very low Fe conditions, several taxa, including *Roseburia*, *[Eubacterium] ectale*, *Clostridium* Cluster IV members, and *Bacteroides* were decreased, while members of the *Lactobacillus* and Enterobacteriaceae family were increased, consistent with a decrease of SCFA, namely butyrate and propionate [96]. 

Experimental animal studies further supported the findings in human studies. These studies pointed out the similar usual suspects, such as elevated abundance of the *Lactobacillus, Enterobacteriaceae* family as well as *Enterococcus* and reduced abundance of *Bacteroides* and *Roseburia* members in iron-deprived mice and young Sprague Dawley rats [97,98]. Besides, relatively low numbers of total anaerobes in the colons of iron-supplemented mice suggested that the provision of Fe(III) suppressed bacteria, likely by the oxidation of normally reduced environments [97]. In a study where researchers only assessed Bacteroidetes, the Enterobacteriaceae family, and Firmicutes, the influence of ferric iron on gut microbiota was investigated, but no effect was found [99]. In rats, iron dose and a time-dependent study showed changes in these usual suspects with addition of changes in *Clostridium difficile* enterotoxin [100]. In a further study with a genetic modification of iron metabolism in mice, the relative abundance of five lactic acid bacteria were significantly different among the mouse lines, suggesting that the deletion of iron metabolism-related genes in the host can affect the intestinal gut composition [101]. It was also shown that a heme-rich diet decreased gut microbial diversity. Major taxonomic changes included an increase in the relative abundance of Proteobacteria, and a decrease in the abundance of Firmicutes, similar to Dextran Sulfate Sodium (DSS)-induced colitis [102]. Additionally, the intestinal lumen may support the growth of bacteria-coding genes that are related to heme uptake and release from RBCs. In return, gut microbiota can play a critical role on iron absorption, as shown in a study in which metabolic changes due to prebiotic administration affected iron absorption [75] via increasing the expression of iron regulatory genes in the colon and duodenum, and an increase of Lactobacillaceae in the colon [103,104]. Further, a study with GF rats showed a decrease in iron uptake compared to SPF mice, as mentioned before [77]. Among all these studies, another important finding is that concentrations of SCFA and branched chain fatty acids (BCFAs; isobutyrate and isovalerate) were altered in adult fecal microbiota and during in vitro experimentation [98,105]. Specifically, low levels of butyrate and propionate were observed during a luminal iron deficiency condition in rats, and luminal iron absorption might be enhanced by *Propionibacteria* via the biosynthesis of propionate [106]. 

Not surprisingly, iron can promote the replication and virulence of gut enteric pathogens including *Salmonella, Shigella*, and *Campylobacter* (Figure 3). Iron availability in the colon lumen is a critical signal for the expression of virulent genes by pathogens and hosts. It has been shown that a ferroportin-mediated efflux of iron, and consequent changes in the amounts of available iron to *Salmonella typhimurium* can decrease the expression of the protein, favoring the growth of this pathogen [107]. This observation was also investigated with different organisms residing in macrophages, and it was supported with the general notion that cellular iron concentration is one of the critical determinants for infectivity [108,109]. Besides the impact of iron availability to pathogens, hepcidin-mediated iron sequestration also influences the host immune response by altering macrophage cytokine production and function [110]. An in vitro study demonstrated that moderate extracellular iron levels can give an advantage for invasion to *Salmonella* when it is cultured with intestinal epithelial cells [105]. Furthermore, the survival of this enteric pathogen in the host cell may partly depend on the host iron status. However, iron does not always elevate the viability and virulence of pathogens. A recent study with a *Citrobacter* infection experimental mouse model showed that dietary iron supplementation induced insulin resistance and increased glucose levels in the intestine that help to suppress the pathogenicity of this bacterium. Additionally, dietary iron was able to drive the selection of attenuated *Citrobacter* strains that can transmit and asymptomatically colonize naive hosts [111]. In general, iron availability in the gut can have a large impact on the infection cycle of a pathogen. The increased luminal iron and intracellular iron in enterocytes may exaggerate or reduce the virulence of enteric pathogens. So far, relatively little is known about a potential link between iron and intestinal infection, and more research is needed to investigate these concepts in detail.

Overall, oral iron intake can influence the gut microbiota of young and adult populations in the short-term. However, we have still no idea of what is the potential effect of oral iron supplementation in a long-term view for health and gastrointestinal-related infection problem. Given the importance of the microbiota in shaping the development and function of the intestinal immune system [17,18,19], iron-dependent changes in gut microbiota could have an impact on infant health and mucosal immune responsiveness, which need to be further investigated with a larger perspective, with randomized controlled trials in human patients yielding concrete clinical outcomes.

## 6. Iron and Inflammatory Bowel Disease (IBD)

Dysbiosis, or imbalance of the gut microbial consortia disrupting their mutualism with the host, may cause intestinal or systemic pathology, including chronic inflammatory bowel disease (IBD) [112,113,114]. Crohn’s disease (CD) and ulcerative colitis (UC) are the two main forms of IBD, each with an annual incidence of 10–30 per 100,000 in Europe and North America, and they are usually diagnosed before age of 35. These are relapsing-remitting immune-mediated, chronic inflammatory intestinal diseases, each with very diverse sub-phenotypes and heterogeneous responsiveness to treatment [28,115]. Unfortunately, no treatment is satisfactory in about 30% of patients, leaving life-long morbidity, malnutrition, and risk of malignancy. Among many complications of the disease, anemia is the most common one and one third of IBD patients suffer from recurrent anemia. It is a condition that develops when the human system lacks either enough healthy red blood cells or hemoglobin. Many people carry on their lives without knowing that they have iron deficiency anemia. Therefore, people are likely to experience symptoms for years without ever knowing the reason behind them [116]. Iron deficiency anemia (IDA) and anemia of chronic disease (ACD) are the most common causes of anemia in these patients, and they often occur simultaneously. Chronic bleeding in the GIT or unbalanced iron absorption/iron homeostasis due to increased systemic hepcidin levels in the presence of ongoing inflammation are the main reasons behind iron deficiency [8,117,118]. This has tremendous impact on the quality of life of IBD patients. Chronic fatigue is commonly instigated by anemia, and it may debilitate patients as much as abdominal pain or diarrhea. The ultimate therapeutic goal is to improve the patient’s quality of life by changing the hemoglobin concentration and iron level in those patients [119].

Iron absorption is down-regulated in IBD patients with the active disease, but it is normal in quiescent IBD patients [120]. Patients with the active disease generally require iron supplementation. However, one should be cautious with oral iron supplementation, which often leads to gastrointestinal side effects such as nausea, abdominal pain, and diarrhea. Several experimental animal model studies using transgenic models or chemically induced colitis suggested that oral iron administration could exacerbate intestinal inflammation [121,122,123,124,125]. Mechanistically, this might be due to ferrous forms of oral iron appearing to be poorly absorbed, and the iron-induced production of reactive oxygen species (ROS) within the lumen of the gut, or the increased growth of pathobionts in the GIT that thrive on iron and inflammation (Figure 3). It is well-characterized that the gut microbiota of IBD patients are relatively different than non-IBD subjects, mostly with an increase of enteropathogenic strains, as shown by many different groups [126,127,128,129,130,131,132]. Dietary iron supplementation leads to disease exacerbation and a higher risk of infection, and an increased abundance of Enterobacteriaceae. Additionally, it has been shown that the absence of luminal ferrous iron was associated with key changes in the intestinal microbiota [125]. Many animal studies that we have also mentioned in Section 5 support the idea that microbial differences might be enlarged upon iron supplementation into the gut. 

In contrast, intravenous iron therapy offers effective alternative management for iron deficiency anemia, since it does not cause side effects and it is more efficient in restoring the iron status in patients [133]. This generally is preferred when iron deficiency co-exists with anemia in clinically active IBD patients. Direct administration of iron into the circulation requires formulations to prevent the cellular toxicity of iron salts, and hence, intravenous iron is usually administered as ferric gluconate, iron sucrose, iron dextran, and ferric carboxymaltose. A study with the intravenous administration of ferric carboxymaltose showed that this therapy was found to be effective and well-tolerated in IBD patients with iron deficiency [134]. In a complementary study in which iron was supplemented either orally or intravenously, the researchers analyzed the effect of iron supplementation of the gut microbiota and metabolites of IBD patients. Even though the route of supplementation did not affect the species richness in the gut, oral iron changed the abundance of *F. prausnitzii* and *Bifidobacterium* [135]. Metabolically, high levels of phosphatidylglycerol (PG), palmitate, and its derivatives in the orally iron-supplemented group were observed, whereas bile acids, tetrahydrodeoxycorticosterone, and other cholesterol derivatives were the characteristics of the intravenously iron-supplemented group [135]. This study identified that CD patients were more prone to iron-supplemented therapy shifts, and oral, but not intravenous, iron therapy affected the presence of specific bacterial species and their products.

Nowadays, there are many good reasons to pay careful attention to iron metabolism than ever before, when dealing with specifically IBD patients with anemia. Until we find a better treatment to IBD, the primary goal is the optimization of supportive care to enhance the patient’s quality of life. To do that, we need to better understand the fine-tuned balance between iron metabolism and microbial population residing in the gut of IBD patients. 

## 7. Iron and Colorectal Cancer

Iron is a limiting factor of growth for many pathobiont bacteria. Contrary, it can also promote a shift in the ratio between pathobionts and gut commensals, with an increase in specific metabolites and inflammation in the intestines. Therefore, a high concentration of iron in the colon leads us to question whether or not iron might also be involved in the initiation or promotion of colonic diseases, specifically colorectal cancer. Despite recent advances in cancer treatment, colorectal cancer still remains one of the deadliest cancer types, with a significantly increased incidence in developing countries with Westernized lifestyles. The incidence of colorectal cancer differs broadly between diverse human populations. It has been suggested that dietary fiber content is of utmost importance, and that it is inversely related to the occurrence of colonic cancer. Since Graf and Eton’s editorial comment in 1985, multiple factors that drive the progression from healthy mucosa to colorectal carcinoma have been identified [136,137]. Accumulating evidences with many in vitro studies and in vivo interventions have consistently supported the role of iron in colorectal cancer risk via a mechanism of increased oxygen radical synthesis and the role of phytic acid, a potent inhibitor of iron-mediated generation of the hazardous oxidant, hydroxy radicals, reversing the augmentation of tumor risk [138,139,140]. 

A majority of the strongest studies confirm that both dietary iron and iron storage augment colorectal cancer risk, as reviewed in these manuscripts [139,141]. A positive association between iron storage (transferrin saturation) in the host system due to mutation in human hereditary hemochromatosis (a.k.a. iron overload disorder; a disorder that causes the body to absorb too much iron from the diet, and excess amount of iron is stored in the body’s tissues and organs, particularly the skin, heart, liver, pancreas, and joints) gene (C282Y mutation), and the development of precancerous lesions in the colon, colonic adenomas, or polyps were reported [142,143]. Additionally, five prospective human cohort studies, including the data of 566,607 individuals and 4,734 cases of colon cancer, showed that a high intake of heme iron was linked with an increased risk of colon cancer, even though one cohort did not identify any association [144,145,146,147,148]. Yet, many critical studies hint on the significant role of diet as a major player in colorectal cancer development [149]. Even though the hemochromatosis gene probably does not play a major role in the majority of colorectal cancers, two different fields of research, genetic and nutritional oncology, have united to find out the mechanisms that drive this type of cancer. The findings that intraluminal iron via interactions with intestinal microbes, promotes of hydroxy radicals, brings the gut microbiota, the hot subjects over the last 5–6 years, to this unity as a third key factor, and shift recent investigations in the microbiota field, which have been largely driven by advances in DNA sequencing (particularly of highly conserved hyper-variable regions of the 16S ribosomal RNA genes in bacteria).

Recent reports showed that *Bacteroides/Prevotella, Clostridum, Streptococcus bovis,* and *Enterococcus faecalis* can produce genotoxic metabolites, such as hydrogen sulphide and secondary bile salts, which likely promote inflammation and carcinogenesis [150,151,152,153]. In defence, *B. longum* and *L. acidophilus* are gut-protective commensals [154,155]. They form a protective barrier against colonization by pathogenic bacteria, and they produce butyrate that act as an anti-carcinogenic agent [156]. Additionally, strains of *Bifidobacteriaceae* family can affect free radical formation by binding iron to their surface, and they promote daily renewal of the colon epithelium, while strains of *Lactobacillus* can reduce the mutagenic effect of bile acids [154,155]. Moreover, antibiotic-based clearance of gut pathobionts reduced the incidence of colon cancer, and altered gut microbiota in mice [157]. These findings were supported with human studies. Advanced colorectal adenoma or carcinoma patients were shown to be deficient in lactic acid-producing commensals [158]. Whether reverting this microbial profile in the patient’s gut might have an effect on disease progression is the one burning question, and even though gut microbiota-dependent dietary changes are promising against colorectal carcinoma, these methods still require further investigation.

## 8. Concluding Remarks

Iron deficiency is a globally serious problem, and it can be corrected to avoid any serious health issues in individuals suffering from it. In this review, we discussed the multi-faceted effects of iron, its administration, and its role on host–microbiota interaction(s) in health and disease (Figure 3). So far, we have a clear view that oral iron administration may impact the gut microbiota profile, and it is the main preferable therapy, even though this has serious gastrointestinal problems including diarrhea, morbidity, and mortality in children, mainly in Africa. From this, the “chicken–egg” question arises, as scientists struggle to find better explanations for iron homeostasis based on iron-dependent fluctuations in the host response, and the growth of gut bugs in the presence of inflammation. It is likely that intestinal microbiota and iron homeostasis are the key parts, but not the only parts, of a more complex interplay that triggers the inflammatory response in the intestines, which can lead to IBD or colorectal cancer. Impressive advancements have been made during the past few years in biomedical science and computation biology, and we are now at a level of better characterization of gut microbiota-dependent inflammatory responses and its direct connection to iron metabolism. Until today, many human studies have only reported observed correlations, and more work is necessary to prove a causal relationship between iron-gut bacteria interactions and the development of gut inflammatory diseases and colorectal cancer. Experimental animal models have assisted in understanding how the gut microbiota interact with excessive amounts of unabsorbed luminal iron, and modern iron therapeutic administration methods for iron deficient populations [159].

## Abbreviations

**ACD**	Anaemia of chronic disease
**BCFA**	Branched chain fatty acids
**BMPs**	Bone morphogenetic proteins
**CD**	Crohn’s disease
**DCYTB**	Duodenal Cytochrome B
**DMT1**	Divalent Metal Transporter 1
**GF**	Germ-free
**GI**	Gastrointestinal
**H_2_O_2_**	Hydrogen peroxide
**HEPH**	Hephaestin
**HMOX1**	Heme Oxygenase 1
**IBD**	Inflammatory bowel disease
**IBS**	Irritable bowel syndrome
**IDA**	Iron deficiency anemia
**Nramp1**	Natural Resistance-Associated
**PG**	Phosphatidylglycerol
**RBC**	Red blood cell
**ROS**	Reactive oxygen species
**SCFA**	Short-chain fatty acids
**SLC40A1**	Solute Carrier Family 40 Member 1
**SLC46A1**	Solute Carrier Family 46 Member 1
**SNP**	Single nucleotide polymorphisms
**SOD**	Superoxide dismutase
**SPF**	Specific pathogen-free
**UC**	Ulcerative colitis
